# Supplementary material for: Identification of photoperiod-induced specific miRNAs in the adrenal glands of Sunite sheep (Ovis aries)
Source: Front Vet Sci. 2022 Jul 22;9:888207. doi: 10.3389/fvets.2022.888207 (PMC9354845; doi:10.3389/fvets.2022.888207)
Supplement: Supplementary file 1 [file Data_Sheet_1.ZIP › Supplementary file/miRDeep2 file.zip!/miRDeep2 file/Novel_Structure/SP42A3/Novel_402.pdf]

| | | |
|---|---|---|
| Provisional ID | : | NC_019458.2_2200 |
| Score total | : | 1.9 |
| Score for star read(s) | : | -1.3 |
| Score for read counts | : | 0 |
| Score for mfe | : | 1.6 |
| Score for randfold | : | 1.6 |
| Score for cons. seed | : | |
| Total read count | : | 111 |
| Mature read count | : | 111 |
| Loop read count | : | 0 |
| Star read count | : | 0 |

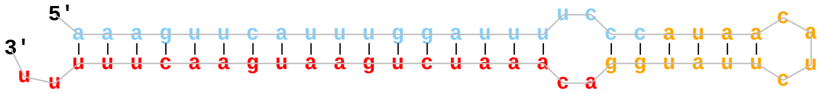


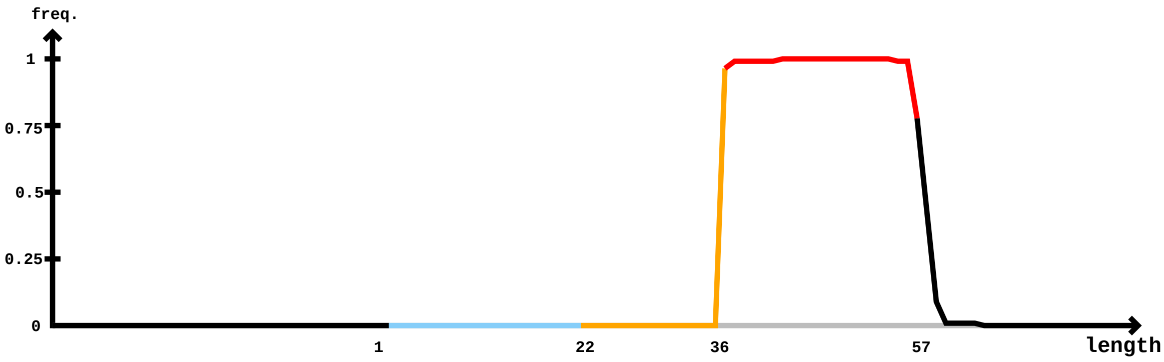


Star Mature

```
5'- cccuaaaccauguaauaucaauugaguuggcaaaaaguucauuuggauuuucccauaacaucuuauggacaaaucugaaugaacuuuuugaccaacccagcagaaucuaga   -3'    exp
    .................((..(((.(((((((((((((((((((((((((..((((((....))))))..)))))))))))))))))))).))))).)))..)).......    reads    mm    sample
    ....................................................................aAaaaucugaaugaacuu.........................    1        1     seq
    ....................................................................aAaaaucugaaugaacuuuu.......................    24       1     seq
    ....................................................................aAaaaucugaaugaacuuuuu......................    69       1     seq
    ....................................................................acaaaucugaaugaacuuuuu......................    5        0     seq
    ....................................................................aAaaaucugaaugaacuuuuuga....................    9        1     seq
    .....................................................................caaaucugaaugaacuuuuu......................    3        0     seq
    ..........................................................................cugaaugaacuuuuugaccaC................    1        1     seq
```
